# Nerve growth factor promotes lysyl oxidase-dependent chondrosarcoma cell metastasis by suppressing miR-149-5p synthesis

**DOI:** 10.1038/s41419-021-04392-2

**Published:** 2021-11-23

**Authors:** Huey-En Tzeng, Syuan-Ling Lin, Louis Anoop Thadevoos, Ming-Yu Lien, Wei-Hung Yang, Chih-Yuan Ko, Chih-Yang Lin, Yu-Wen Huang, Ju-Fang Liu, Yi-Chin Fong, Hsien-Te Chen, Chih-Hsin Tang

**Affiliations:** 1 Ph.D. Program for Cancer Molecular Biology and Drug Discovery, College of Medical Science and Technology, Taipei Medical University, Taipei, Taiwan; 2 Graduate Institute of Cancer Biology and Drug Discovery, College of Medical Science and Technology, Taipei Medical University, Taipei, Taiwan; 3 Division of Hematology/Oncology, Department of Medicine, Taipei Medical University Hospital, Taipei, Taiwan; 4 School of Medicine, College of Medicine, Taipei Medical University, Taipei, Taiwan; 5 Translational Medicine Research Center, China Medical University Hospital, Taichung, Taiwan; 6 International Master Program of Biomedical Sciences, China Medical University, Taichung, Taiwan; 7 Division of Hematology and Oncology, Department of Internal Medicine, China Medical University Hospital, Taichung, Taiwan; 8 School and Medicine, China Medical University, Taichung, Taiwan; 9 School of Chinese Medicine, China Medical University, Taichung, Taiwan; 10 Department of Nursing, National Taichung University of Science and Technology, Taichung, Taiwan; 11 Department of Orthopedic Surgery, Taichung Hospital, Ministry of Health and Welfare, Taichung, Taiwan; 12 Department of Orthopedic Surgery, China Medical University Hospital, Taichung, Taiwan; 13 Department of Pharmacology, School of Medicine, China Medical University, Taichung, Taiwan; 14 Graduate Institute of Biomedical Sciences, China Medical University, Taichung, Taiwan; 15 School of Oral Hygiene, College of Oral Medicine, Taipei Medical University, Taipei, Taiwan; 16 Department of Sports Medicine, College of Health Care, China Medical University, Taichung, Taiwan; 17 Department of Orthopedic Surgery, China Medical University Beigang Hospital, Yunlin, Taiwan; 18 Department of Biotechnology, College of Health Science, Asia University, Taichung, Taiwan; 19 Chinese Medicine Research Center, China Medical University, Taichung, Taiwan

**Keywords:** Bone cancer, Cell invasion

## Abstract

Chondrosarcoma is a malignancy of soft tissue and bone that has a high propensity to metastasize to distant organs. Nerve growth factor (NGF) is critical for neuronal cell growth, apoptosis, and differentiation, and also appears to promote the progression and metastasis of several different types of tumors, although the effects of NGF upon chondrosarcoma mechanisms are not very clear. We report that NGF facilitates lysyl oxidase (LOX)-dependent cellular migration and invasion in human chondrosarcoma cells, and that NGF overexpression enhances lung metastasis in a mouse model of chondrosarcoma. NGF-induced stimulation of LOX production and cell motility occurs through the inhibition of miR-149-5p expression, which was reversed by PI3K, Akt, and mTOR inhibitors and their respective short interfering RNAs. Notably, levels of NGF and LOX expression correlated with tumor stage in human chondrosarcoma samples. Thus, NGF appears to be a worthwhile therapeutic target for metastatic chondrosarcoma.

## Introduction

Chondrosarcoma is a common malignancy that occurs typically in cartilage-enriched bone (e.g., femur, tibia, or pelvis) [1, 2] that displays a high propensity to metastasize to distant organs [1]. High-grade chondrosarcomas are particularly prone to metastasize to the lungs [3, 4], so delaying or inhibiting this phenomenon is essential for patient survival. Metastasis of any tumor is characterized by the secretion of proteolytic enzymes such as matrix metalloproteinases (MMPs) and lysyl oxidase (LOX), capable of degrading the extracellular matrix (ECM) and basement membrane [5, 6]. LOX is an extracellular cuproenzyme necessary for the catalyzation of collagen and elastin crosslinking in the ECM [7]. Several different tumors, such as oral, breast, and gastric cancers, overexpress LOX [8, 9], while increased LOX expression in premalignant host tissue is associated with a higher tumor incidence and burden [10]. Moreover, the alteration of the ECM by LOX-induced catalyzing of collagen and elastin crosslinkages is thought to be one way in which LOX promotes metastasis: this alteration of the ECM helps to form microenvironments that recruit nonmalignant host cells into the primary tumor microenvironment, and these alterations to the local microenvironments enable premetastatic “permissive niches” to emerge that allow colonization of tumor cells and formation of secondary metastases [11]. Thus, inhibiting LOX expression would appear to be a useful therapeutic tactic in tumor metastasis.

MicroRNAs (miRNAs) are involved in the cellular processes of many human diseases, including cancers, by regulating different activities of the tumor cell, including apoptosis, proliferation, angiogenesis, drug resistance, and metastasis [12]. The importance of miRNAs in tumorigenesis is underlined by the fact that they target key metabolic enzymes and protein messenger RNAs (mRNAs) [13]. In particular, researchers have suggested that miRNA mimics or inhibitors of metabolic processes and gene regulatory events could improve overall survival in lung cancer [13]. Moreover, miRNA levels may serve as potential biomarkers and therapeutic targets in cancers [14]. Notably, miRNA levels of LOX are regulated by miR-149 [15], while miR-29 contributes to the maintenance of ECM homeostasis by adversely regulating ECM protein production [15, 16].

Nerve growth factor (NGF) is vital for neuronal cell growth, apoptosis, and differentiation [17]. The binding of NGF to its receptor tropomyosin receptor kinase A (TrkA) activates intracellular signaling, immune cell proliferation, differentiation, and survival [18]. It has also been suggested that NGF plays an integral part in the progression of several types of malignancies, such as ovarian, prostate, and liver cancers [19–21], as well as metastasis in various tumors [21–23]. We have previously reported that NGF promotes MMP-2 expression and chondrosarcoma cell migration by inhibiting miR-423-5p expression through the FAK and c-Src signaling cascades [24]. We therefore set out to define how NGF could affect chondrosarcoma metastasis, in in vitro and in vivo explorations. We also sought to determine whether LOX mediates NGF-induced migration and invasion abilities of chondrosarcoma cells, and whether this process is regulated by PI3K, Akt, and mTOR signaling. Finally, we examined potential candidate miRNAs that could affect LOX mRNA expression in chondrosarcoma cell lines, and the potential role of NGF in this process.

## Results

### NGF promotes LOX-dependent migration and invasion of chondrosarcoma

**Fig. 1:**
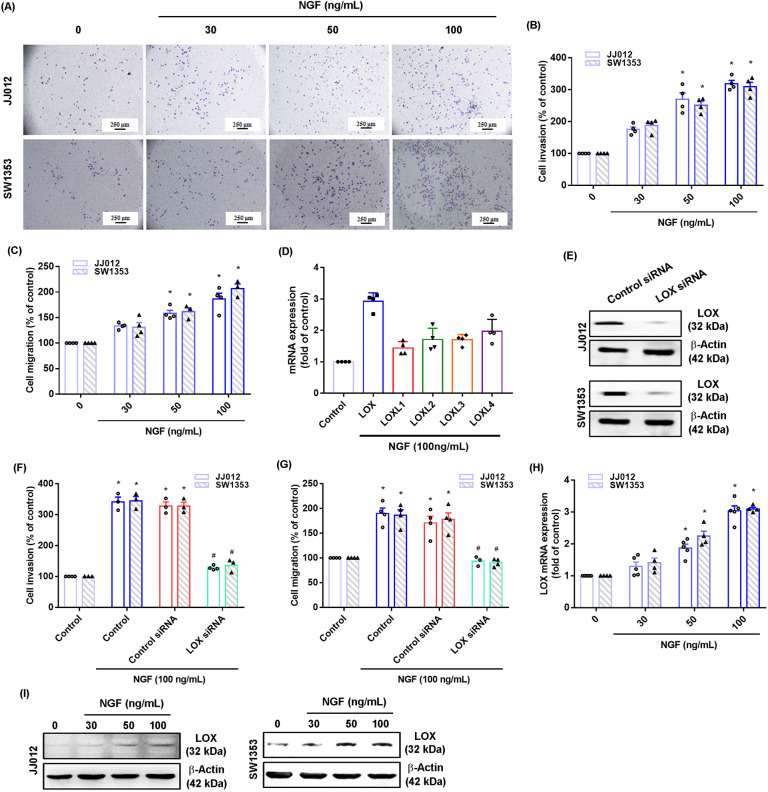
NGF promotes LOX-dependent cell migration and invasion in human chondrosarcoma. **A**–**C** Cells were incubated with NGF (30–100 ng/mL) for 18–24 h and cell invasion and migration was examined by the Transwell assay. **D** JJ012 cells were incubated with NGF (100 ng/mL) for 24 h and qPCR examined the levels of mRNA expression for all LOX family members. **E**–**G** Cells were transfected with LOX siRNAs for 24 h, and then stimulated with NGF for 18–24 h. Cell invasion and migration, as well as levels of LOX expression, were examined by Transwell and western blot assays. (H&I) Cells were incubated with NGF (30–100 ng/mL) for 24 h and levels of LOX mRNA and protein expression were examined by qPCR and western blot assays. *p < 0.05 compared with the control group; ^#^*p* < 0.05 compared with the NGF-treated group.

NGF is associated with cancer cell progression and survival in several different types of cancers [25, 26]. We first investigated the effects of NGF upon cell motility in chondrosarcoma cell lines JJ012 and SW1353. Treatment of chondrosarcoma cells with NGF promoted invasion and migration ability, according to Transwell assay data (Fig. 1A–C). LOX family members (LOX, LOXL1, LOXL2, LOXL3, LOXL4) reportedly mediate cell migration and metastasis of different cancers [11, 27]. We therefore examined whether the LOX family plays a role in NGF-induced migration and invasion in chondrosarcoma. Stimulating cells with NGF enhanced LOX family mRNA expression and LOX mRNA by the greatest amount (Fig. 1D). Transfection of cells with LOX siRNA antagonized NGF-induced promotion of migration and invasion (Fig. 1E–G). Stimulation of cells with NGF enhanced LOX mRNA and protein expression (Fig. 1H, I), implying that LOX is critical to NGF-promoted chondrosarcoma cell migration and invasion.

### PI3K, Akt, and mTOR signaling regulates NGF-induced promotion of LOX-mediated chondrosarcoma cell migration and invasion

**Fig. 2:**
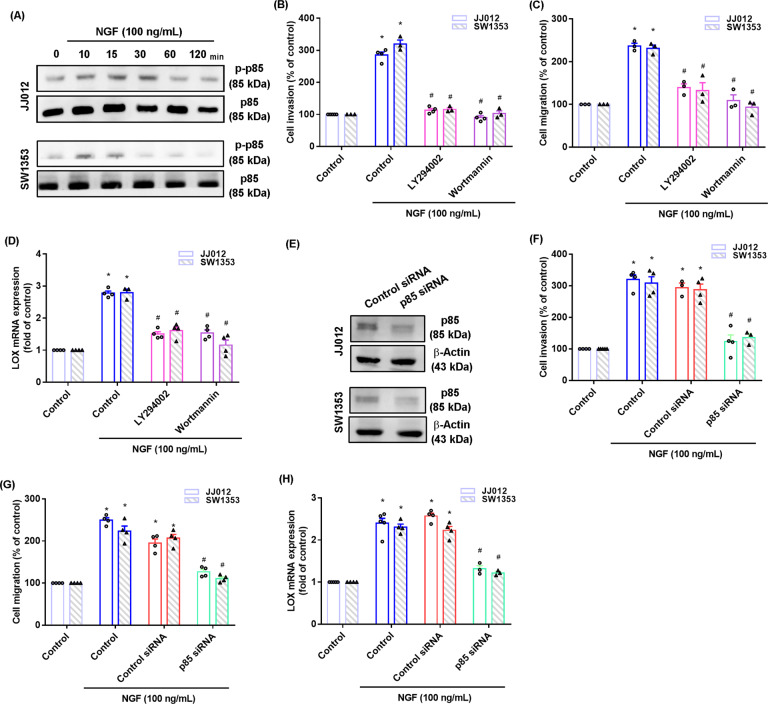
The PI3K pathway mediates NGF-induced LOX expression and cell motility. **A** Cells were incubated with NGF for the indicated time intervals; p85 phosphorylation was examined by western blot. **B**–**D** Cells were treated with PI3K inhibitors (Ly2942002 and wortmannin) for 30 min, and then stimulated with NGF for 18–24 h. Cell migratory and invasive activities, as well as levels of LOX expression, were examined by Transwell and qPCR assays. **E** Cells were transfected with a p85 siRNA and p85 expression was examined by western blot. **F**–**H** Cells were transfected with a p85 siRNA for 24 h, and then stimulated with NGF for 18–24 h. Cell invasion and migration and levels of LOX expression were examined by Transwell and qPCR assays. *p < 0.05 compared with the control group; ^#^*p* < 0.05 compared with the NGF-treated group.

**Fig. 3:**
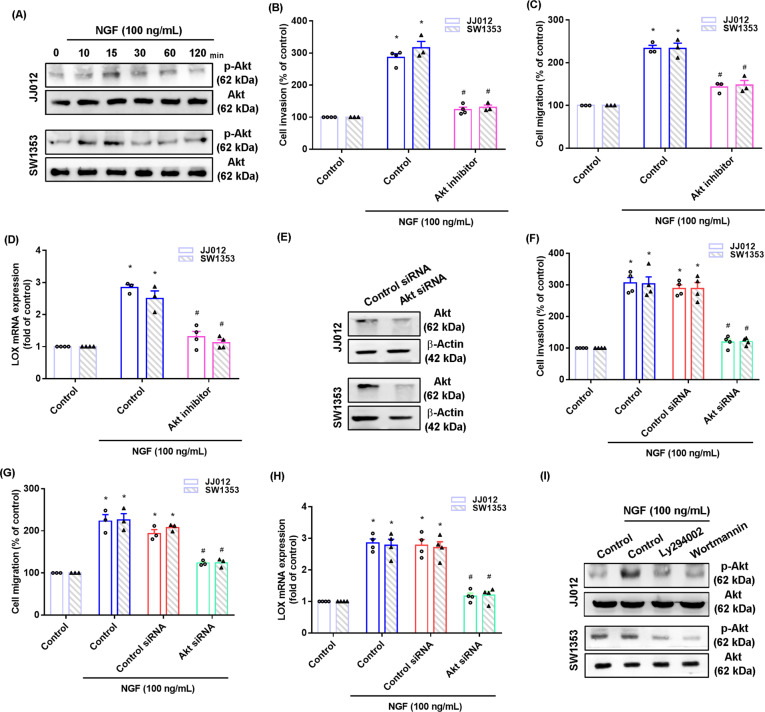
The Akt pathway mediates NGF-induced LOX expression and cell motility. **A** Cells were incubated with NGF for the indicated time intervals; Akt phosphorylation was examined by western blot. **B**–**D** Cells were pretreated with a Akt inhibitor (Akt i) for 30 min, and then stimulated with NGF for 18–24 h. Cell invasion and migration and levels of LOX expression were examined by Transwell and qPCR assays. **E** Cells were transfected with a Akt siRNA and Akt expression was examined by western blot. **F**–**H** Cells were transfected with a Akt siRNA for 24 h, and then stimulated with NGF for 18–24 h. Cell migratory and invasive activities, as well as levels of LOX expression, were examined by Transwell and qPCR assays. I Cells were pretreated with PI3K inhibitors for 30 min and then stimulated with NGF for 15 min; Akt phosphorylation was examined by western blot. *p < 0.05 compared with the control group; ^#^*p* < 0.05 compared with the NGF-treated group.

PI3K and Akt signaling helps to drive chondrosarcoma metastasis [28, 29]. NGF stimulation time-dependently induced p85 and Akt phosphorylation in both cell lines (Figs. 2A and 3A). Treating cells with PI3K inhibitors (Ly294002 and wortmannin) or an Akt inhibitor (Akt i) significantly reduced NGF-enhanced promotion of cell motility and LOX synthesis (Figs. 2B–D and 3B–D). Similar effects were observed when the chondrosarcoma cells were transfected with p85 or Akt siRNAs (Figs. 2E–H and 3E–H). PI3K inhibitor treatment antagonized NGF-facilitated Akt phosphorylation (Fig. 3I), indicating that the PI3K/Akt signaling pathway controls NGF-enhanced LOX synthesis, as well as chondrosarcoma cell migration and invasion.

**Fig. 4:**
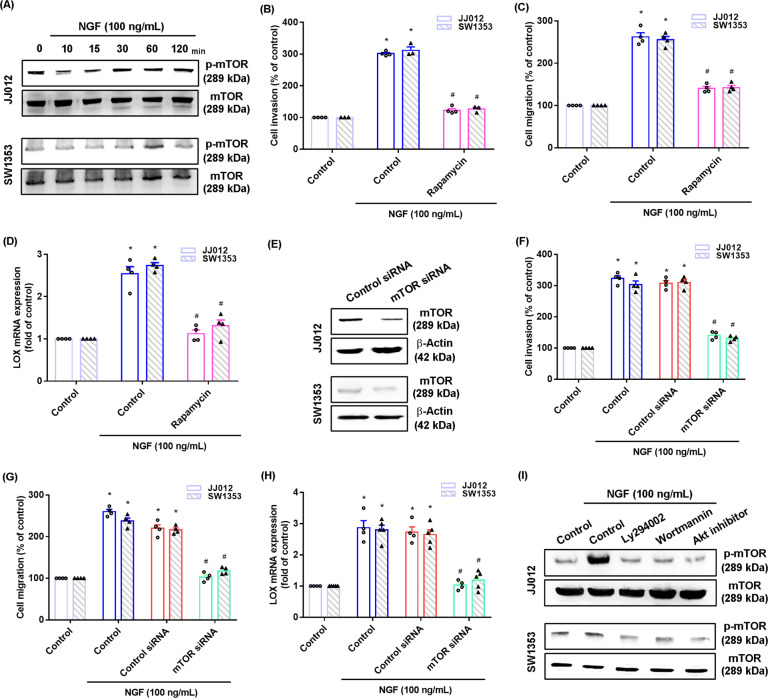
PI3K/Akt-dependent mTOR activation mediates NGF-induced LOX expression and cell motility. **A** Cells were incubated with NGF for the indicated time intervals; mTOR phosphorylation was examined by western blot. **B**–**D** Cells were pretreated with an mTOR inhibitor (rapamycin) for 30 min, and then stimulated with NGF for 18–24 h. Cell migratory and invasive activities, as well as levels of LOX expression, were examined by Transwell and qPCR assays. **E** Cells were transfected with an mTOR siRNA and mTOR expression was examined by western blot. **F**–**H** Cells were transfected with a mTOR siRNA for 24 h, and then stimulated with NGF for 18–24 h. Cell migratory and invasive activities, as well as levels of LOX expression, were examined by Transwell and qPCR assays. **I** Cells were pretreated with PI3K and Akt inhibitors for 30 min, and then stimulated with NGF for 60 min; mTOR phosphorylation was examined by western blot. *p < 0.05 compared with the control group; ^#^*p* < 0.05 compared with the NGF-treated group.

The PI3K/Akt signaling pathway regulates downstream mTOR activity and together they control the metastatic potential of chondrosarcoma [28]. Treatment of chondrosarcoma cell lines with NGF upregulated mTOR phosphorylation (Fig. 4A). Incubation of cells with the mTOR inhibitor rapamycin reduced the effects of NGF upon cell migration and invasion, as well as LOX synthesis (Fig. 4B–D). Transfection of the cells with an mTOR siRNA confirmed the similar effects of NGF (Fig. 4E–H). In addition, PI3K and Akt inhibitors reversed NGF-mediated mTOR phosphorylation (Fig. 4I), suggesting that PI3/Akt-dependent mTOR activation mediates NGF-promoted increases in LOX expression and cell motility.

### The miR-149-5p/LOX axis regulates NGF-enhanced stimulation of chondrosarcoma cell migration and invasion

**Fig. 5:**
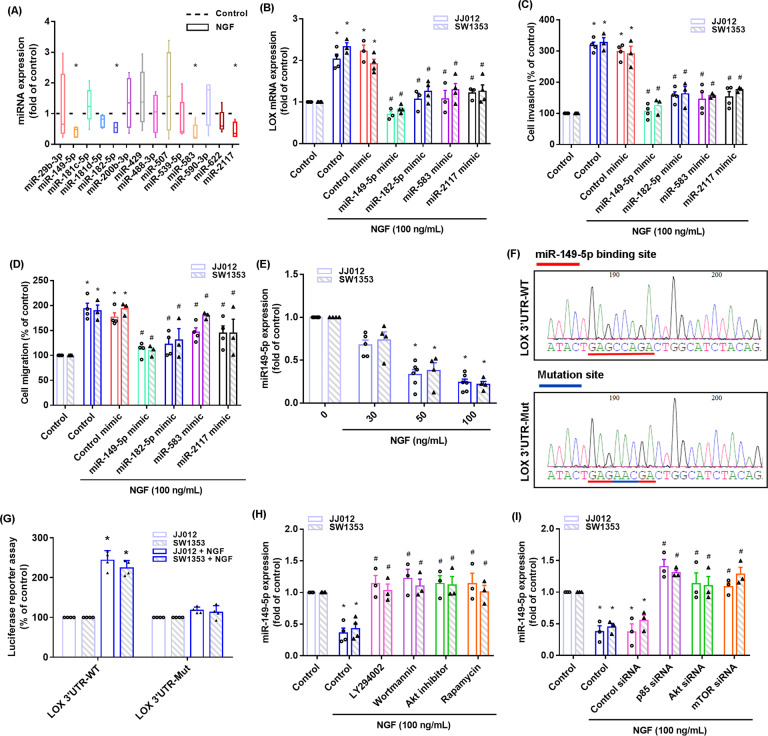
Inhibition of miR-149-5p mediates NGF-induced promotion of LOX expression and motility of human chondrosarcoma cells. **A** MiRNA target prediction software was used to identify miRNAs that potentially bind to the LOX 3′-UTR. **B**–**D** Cells were transfected with miR-149-5p, miR-183-5p, miR-583, and miR-2117 mimics for 24 h, and then stimulated with NGF for 18–24 h. Cell migratory and invasive activities, as well as levels of LOX expression, were examined by Transwell and qPCR. **E** SW1353 and JJ012 cells were incubated with NGF for 24 h and miR-149-5p levels were examined by qPCR. **F** The wild type and mutant LOX 3′-UTRs contain the miR-149-5p-binding site. **G** Cells were transfected with 3′-UTR plasmids as indicated, and then stimulated with NGF for 24 h. Luciferase activity was examined. (H&I) Cells were pretreated with PI3K, Akt, and mTOR inhibitors for 30 min, or an siRNA for 24 h, and then stimulated with NGF for 24 h prior to qPCR analysis of miR-149-5p levels. *p < 0.05 compared with the control group; ^#^*p* < 0.05 com*p*ared with the NGF-treated group.

miRNA-associated regulation of LOX expression is a critical mechanism in the progression and metastasis of cancer cells [16, 30]. A search of five online databases (miRWalk, miRanda, miRMap, RNAhybrid, and TargetScan) for miRNA target prediction indicated that the 3′-UTR region of LOX mRNA contains 14 promising candidate miRNAs. Treatment of JJ012 cells with NGF (100 ng/mL) significantly downregulated the expression of four miRNAs (miR-149-5p, miR-183-5p, miR-583, and miR-2117) (Fig. 5A). Transfection of chondrosarcoma cells with the mimics of these four miRNAs significantly reduced NGF-induced stimulation of cell migration and LOX mRNA expression in both chondrosarcoma cell lines; miR-149-5p mimic had the greatest inhibitory effects (Fig. 5B–D). Treatment of JJ012 cells with NGF (30, 50, or 100 ng/mL) obviously inhibited miR-149-5p synthesis in both chondrosarcoma cell lines, in a concentration-dependent manner (Fig. 5E). Analyses of the LOX 3′-UTR luciferase plasmids revealed that NGF increased luciferase activity of the wild-type, but not mutant, LOX 3′-UTRs (Fig. 5F, G). Next, we investigated whether PI3K, Akt, and mTOR signaling regulate NGF-induced suppression of miR-149-5p synthesis. PI3K, Akt, and mTOR inhibitors, and their respective siRNAs, all reversed NGF-induced inhibition of miR-149-5p expression (Fig. 5H, I). These results indicate that miR-149-5p controls LOX expression by anchoring to the 3′-UTR of the human *LOX* gene via the PI3K/Akt/mTOR pathway.

### Overexpression of NGF promotes chondrosarcoma metastasis to lungs in mice

**Fig. 6:**
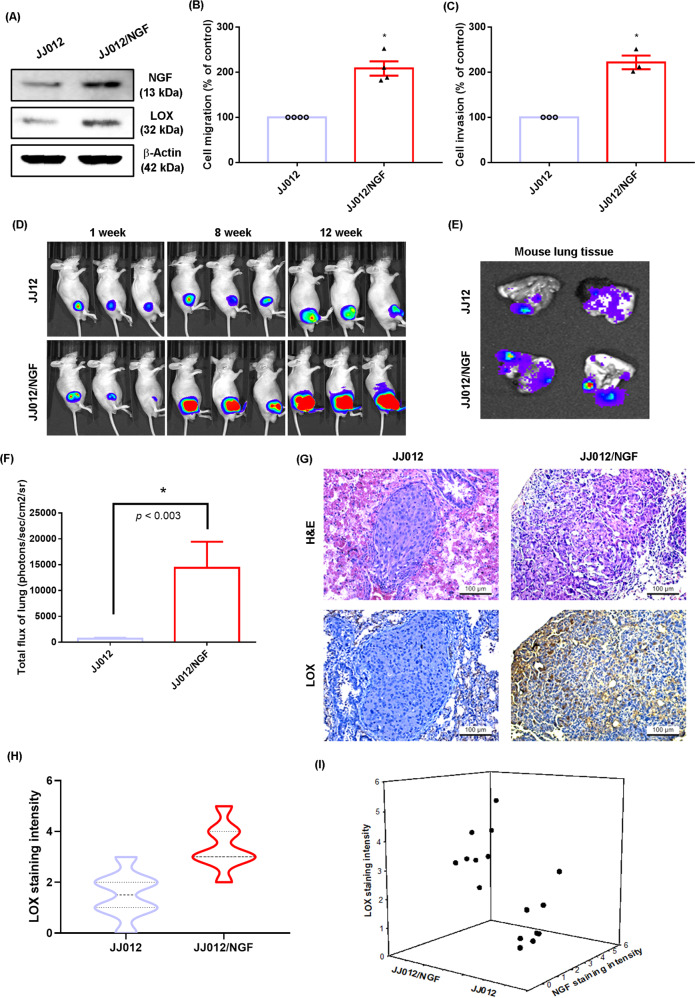
NGF promotes chondrosarcoma metastasis to lungs in vivo. A–**C** NGF and LOX levels, as well as the migratory ability of JJ012 and JJ012/NGF cells, were examined by western blot and Transwell. **D** The mice were injected with JJ012 or JJ012/NGF cells. Lung metastasis was monitored by bioluminescence imaging at the indicated time intervals, and then quantified by photon images. **E**, **F** After 12 weeks, the mice were humanely sacrificed and the lung tissue was excised, photographed, and quantified. **G**, **H** Levels of LOX expression in lung tumors were subjected to IHC analysis. **I** The positive correlation between NGF and LOX protein expression was observed in mice lung tissue. *p < 0.05 compared with the JJ012 group.

In our previous experiments, we used an established orthotopic mouse model of chondrosarcoma lung metastasis to examine the stimulatory effects of NGF in metastatic chondrosarcoma [28], which confirmed that NGF promotes chondrosarcoma metastasis in mouse lungs by stimulating MMP-2 expression [24]. In this study, NGF overexpression in JJ012 cells (JJ012/NGF) promoted high levels of both NGF and LOX protein, which significantly increased the migratory ability of JJ012 cells (Fig. 6A–C). JJ012 and JJ012/NGF cells were orthotopically implanted into the right leg tibia and tumor size was monitored by the IVIS system (Fig. 6D). The IVIS data confirmed that overexpression of NGF significantly increased tumor growth in the tibia (Fig. 6D). After 12 weeks, the IVIS results revealed that metastasis to the lung was significantly more likely with JJ012/NGF cells than with JJ012 cells (Fig. 6E, F). Immunohistochemistry (IHC) results revealed significant increases in the levels of LOX expression in the JJ012/NGF orthotopic model (Fig. 6G, H), confirming that NGF facilitates the metastasis of chondrosarcoma to the mouse lung. Interestingly, the positive correlation between NGF and LOX protein expression was observed in mice lung tissue (Fig. 6I). Our data indicate that endogenous NGF augments LOX-dependent lung metastasis of chondrosarcoma in mice.

### NGF and LOX levels are positively correlated in human chondrosarcoma tissue

**Fig. 7:**
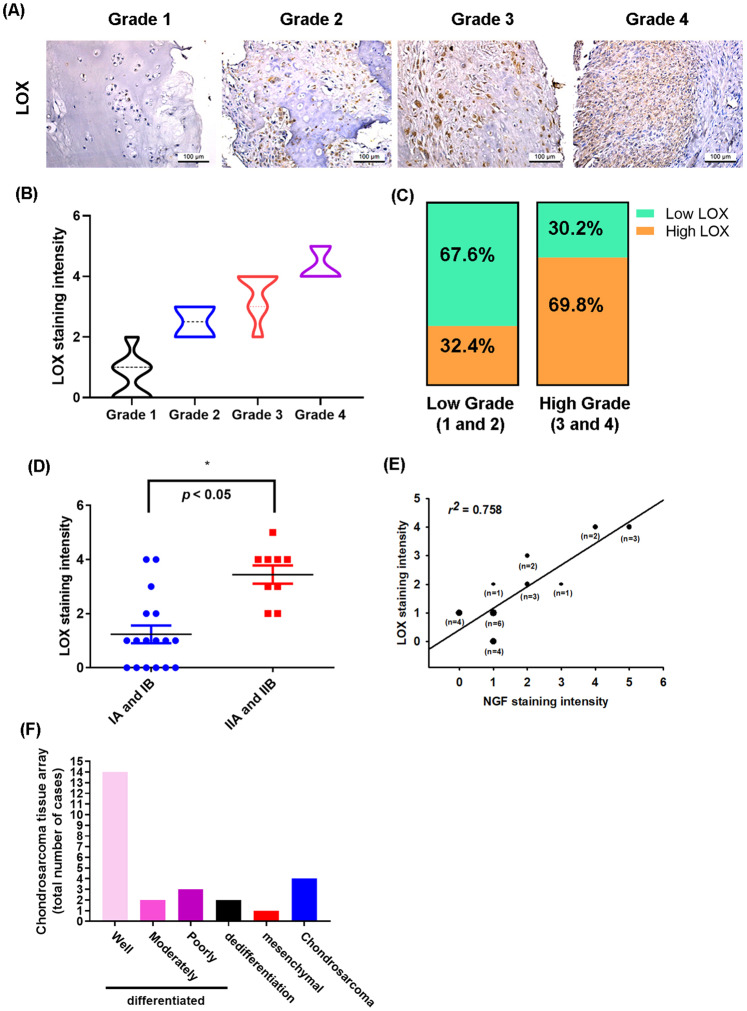
NGF and LOX levels correlated with chondrosarcoma tumor stage. **A**–**D** IHC-stained tissue samples from chondrosarcoma patients were stained with LOX antibodies, then photographed and quantified. **E** Levels of NGF and LOX were positively correlated. **F** The chondrosarcoma tissue array cases of subtypes. *p < 0.05 compared with the early-stage (IA and IB) tumor group.

In previous IHC tissue array investigations, we have documented higher levels of NGF and MMP-2 expression in patients with higher-grade chondrosarcoma than in those with lower-grade disease [24]. We also observed in that study a positive significant correlation between MMP-2 and NGF staining intensities of human chondrosarcoma tissue, indicating an association between the levels of these proteins and the progression of chondrosarcoma disease [24]. In this study, we investigated LOX expression in patients with chondrosarcoma. IHC data revealed higher levels of LOX expression in patients with higher-grade chondrosarcoma than in those with lower-grade disease; the levels of LOX expression were reflected by tumor stage (Fig. 7A–C). These results are quantified in Fig. 7D, which illustrate how the levels of LOX expression were significantly higher in higher-stage tumors (IIA and IIB) than in lower-stage tumors (IA and IB). A positive correlation observed between NGF and LOX staining intensity of human chondrosarcoma tissue (*r*^*2*^ = 0.534, Fig. 7E) indicates that the levels of these proteins (NGF and LOX) are associated with the progression of chondrosarcoma disease. Finally, the chondrosarcoma types tissue array number of cases are shown in Fig. 7F.

## Discussion

Chondrosarcoma is a malignant bone neoplasm that constitutes almost one-third (~26%) of all bone cancers and is characterized by the production of cartilage matrix [31]. Chondrosarcoma is divided into several subtypes: conventional, dedifferentiated, mesenchymal, clear cell, periosteal, and myxoid. The vast majority (85%) are conventional central chondrosarcomas that occur mainly in adulthood and old age, most often in an intramedullary location and involving the bones of the trunk, pelvis, femur, and humerus [32]. Chemotherapy and radiotherapy have very limited effectiveness, so surgery is the major therapeutic modality for chondrosarcoma. This malignancy is notorious for its aggressive clinical course and propensity to metastasize [33]. An effective adjuvant therapy is urgently needed to suppress chondrosarcoma metastasis [1, 3]. NGF plays an important role in tumor cell proliferation, migration, and survival [25, 26]. Our investigation has found that levels of NGF and LOX expression are positively correlated with tumor staging in patients with chondrosarcoma. We also confirmed that NGF facilitates LOX-dependent chondrosarcoma cell migration and metastasis by suppressing miR-149-5p synthesis via PI3K, Akt, and mTOR signaling.

The lysyl oxidase family of enzymes (encoded by LOX and LOXL1–4) catalyze the final reaction required for collagen and elastin crosslinking, an essential step that ensures structural integrity and function of several tissues, including bone [34]. The localization of LOX to the ECM, especially in cancer cells, makes it an attractive target for activated prodrugs that tend to accumulate in the tumor stroma [7]. The crosslinking of collagen and elastin facilitated by LOX enhances the proliferation of tumor cells and promotes metastatic colonization and a fibrotic microenvironment that improves the survival of tumor cells, supporting the development of metastasis [11, 35]. For instance, laminin A4 (a component of the ECM) is associated with metastasis in soft sarcoma cells and is supported by the upregulation of LOX, which remolds the ECM of sarcoma cells [36]. Treatment that targets LOX lessens the severity of fibrosis and also restricts metastatic colonization boosted by fibrosis [35]. Our study is the first to describe an association between levels of LOX expression and tumor stage in chondrosarcoma tissue specimens. Our in vitro and in vivo evidence suggests that NGF facilitates LOX-dependent chondrosarcoma metastasis. LOX is therefore a promising molecular targeting for treating chondrosarcoma metastasis.

Activation of the PI3K/Akt pathway is important for regulating various cellular functions [37]. This signaling pathway also regulates the metastatic potential of human chondrosarcoma [28]. In addition, LOX is involved in the hypoxic upregulation of HIF-1α, while LOX and HIF-1α potentiate each other to foster colon tumor progression through the PI3K/Akt signaling pathway [38]. In our previous study, NGF promoted MMP-2 expression and chondrosarcoma cell migration through the FAK and c-Src signaling cascades [24]. We have also previously found that the pretreatment of chondrosarcoma cells with MEK, ERK, JNK, p38, and PKCα/β inhibitors downregulated NGF-induced stimulation of cell migration (unpublished data). However, these inhibitors will not affect NGF-induced LOX genes expression. Our study results here show that NGF promotes phosphorylation of PI3K and Akt, while PI3K and Akt pharmacological inhibitors suppress NGF-induced promotion of LOX expression, chondrosarcoma cell migration, and invasion. This phenomenon was confirmed by similar effects observed with genetic siRNAs of PI3K and Akt. Previous evidence stating that the PI3K/Akt pathway is an upstream molecule of mTOR, with the capacity to regulate cell motility [39], was confirmed by our data showing that both an mTOR inhibitor and siRNA antagonized NGF-induced LOX production and cell motility. PI3K and Akt inhibitors also inhibited NGF-promoted phosphorylation of mTOR, indicating that PI3K/Akt-dependent mTOR activation mediates NGF-facilitated promotion of LOX synthesis and chondrosarcoma cell motility.

MiRNAs post-transcriptionally regulate gene expression [40]. During tumor metastasis, aberrant miRNA expression mediates cancer cell migration and invasion [41]. Here, our analysis of five open-source databases identified that 14 miRNAs potentially interfere with LOX transcription. We enhanced miR-149-5p levels in chondrosarcoma cells by transfecting them with a specific miR-149-5p mimic, which markedly reduced LOX synthesis and the migratory capacity of the cells. miR-149-5p expression was negatively correlated with LOX expression, as well as with the migratory and invasive activities of chondrosarcoma cells. Thus, our evidence has identified that miR-149-5p exhibits novel antimetastatic properties. In addition, NGF significantly lowered miR-149-5p expression. Treating chondrosarcoma with PI3K, Akt, and mTOR inhibitors reversed NGF-promoted inhibition of miR-149-5p expression, which suggests that NGF may increase LOX expression and chondrosarcoma metastasis by inhibiting miR-149-5p synthesis via the PI3K, Akt, and mTOR signaling cascades. Whether these signaling cascades regulate miR-149-5p expression via transcriptional or post-transcriptional regulation needs further investigation.

Our study is limited by the fact that we had very few samples to adequately represent the various human chondrosarcoma subtypes. That is, our human chondrosarcoma tissue array contained samples from 26 patients; 19 of differentiated chondrosarcoma, 2 of dedifferentiated chondrosarcoma, 1 of mesenchymal chondrosarcoma, and 4 of chondrosarcoma, which did not constitute a sufficiently large enough sample to avoid false-positive conclusions and distinguish differences in protein expression among the different subtypes (Fig. 7F). Moreover, as these tissue arrays do not provide information about neoadjuvant pharmacotherapy, chemotherapy, or radiotherapy, we could not perform any detailed analyses in regard to LOX levels and tumor stage. We therefore recommend using larger clinical samples in any future research.

**Fig. 8:**
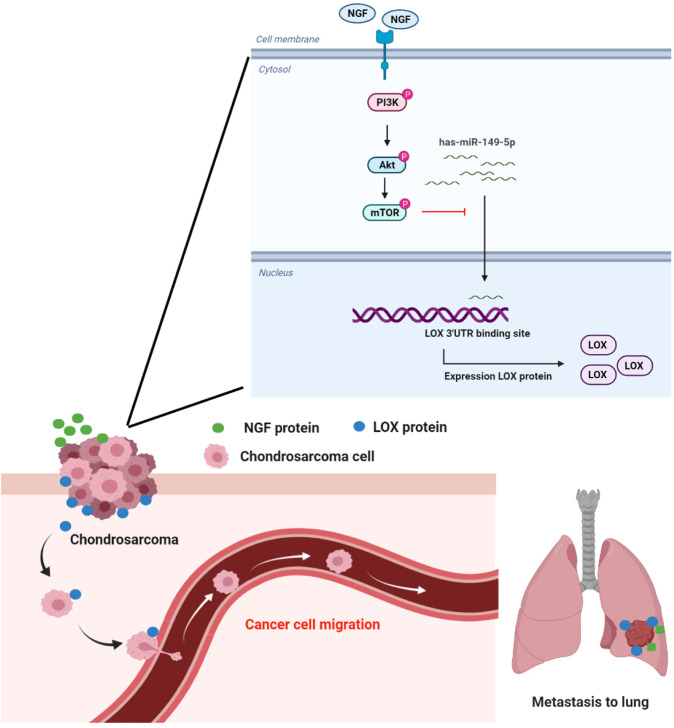
Schema illustrating the effects of NGF in chondrosarcoma metastasis. NGF promotes LOX-dependent metastasis of chondrosarcoma by suppressing miR-149-5p synthesis via the PI3K, Akt, and mTOR signaling pathways.

In conclusion, our study has identified that NGF promotes LOX-dependent migratory and invasive activities in human chondrosarcoma cells by suppressing miR-149-5p synthesis via the PI3K, Akt, and mTOR signaling cascades (Fig. 8). It appears to be worth targeting NGF expression in metastatic chondrosarcoma.

## Materials and methods

### Materials

NGF (SC-365944), LOX (SC-373995), p85 (SC-1637), and Akt (SC-5298) antibodies were purchased from Santa Cruz (Biotechnology, CA, USA). The total mTOR (2983S) and phosphorylated forms of p85 (4228S), Akt (4060S), and mTOR (5536S) antibodies were purchased from Cell Signaling Technology (Danvers, MA, USA). β-Actin (a5441) antibody was purchased from Sigma (Cambridge, MA, USA). All ON-TARGET*plus* short interfering (si)RNAs were obtained from Dharmacon (Lafayette, CO, USA). Quantitative polymerase chain reaction (qPCR) primers and probes, as well as Taqman^®^ One-Step PCR Master Mix, were supplied by Applied Biosystems (Foster City, CA, USA). Recombinant human NGF was obtained from PerpoTech (Rocky Hill, NJ, USA). All other chemicals used in this study were supplied by Sigma-Aldrich (St. Louis, MO, USA).

### Cell culture

The human chondrosarcoma cell line JJ012 was kindly provided by Dr. Sean P. Scully (University of Miami School of Medicine, Miami, FL, USA), while the SW1353 chondrosarcoma cell line was obtained from the American Type Cell Culture Collection (Manassas, VA, USA). JJ012 cells stably expressing the NGF complementary DNA (cDNA) clone (JJ012/NGF cells) were established according to our previous method [28]. Cells were cultured 50%/50% in Dulbecco’s modified Eagle’s medium (DMEM)/alpha-minimum essential medium (α-MEM) medium, 10% fetal bovine serum (FBS), and antibiotics, and then maintained in a humidified incubator at 37 °C in 5% CO_2_.

### Cell migration and invasion assay

Chondrosarcoma cells were seeded into the upper chamber of Transwell plates (Costar, NY, USA) precoated with a layer of Matrigel for the invasion assay. NGF and pharmaceutical inhibitors were added to the lower chamber. After 18 h (migration assay) or 24 h (invasion assay) of incubation, migrated cells were fixed with 3.7% formaldehyde and stained with crystal violet, and then counted manually under the microscope [42, 43].

### Western blot analysis

After the indicated treatments, chondrosarcoma cells were lysed in RIPA buffer. The extracted proteins were resolved by SDS-PAGE and transferred to Immobilon^®^ polyvinylidene fluoride (PVDF) membranes. Western blot analysis was performed using the methodology described in our previous reports [44–46].

### mRNA and miRNA quantification

Total RNA was extracted from chondrosarcoma cells using TRIzol reagent and RNA concentrations were determined using a NanoVue Plus spectrophotometer (GE Healthcare Life Sciences, Pittsburgh, PA, USA). The M-MLV RT kit (Thermo Fisher Scientific, Waltham, MA, USA), and the Mir-X™ miRNA First-Strand Synthesis kit (Clontech, Mountain View, CA, USA) were used to perform reverse transcription of total RNA into cDNA. Quantitative real-time PCR (qPCR) analysis was performed according to our previous reports [47, 48].

### Luciferase assay

The human LOX luciferase reporter plasmids containing wild type or mutant sequences of the three prime untranslated region (3′-UTR) encompassing miR-149-5p-binding sites obtained from MDBio Inc. (Taipei, Taiwan). Chondrosarcoma cells were transfected with the plasmids using Lipofectamine 2000 (Thermo Fisher Scientific, Waltham, MA, USA), and then stimulated with NGF for 24 h. Luciferase activity was monitored using a luciferase assay kit [47, 49, 50].

### Tumor xenograft study

Four-week-old male BALB/c nude mice (8 in each group; randomly assigned) were bought from Taipei’s National Laboratory Animal Center and orthotopically injected with JJ012 or JJ012/NGF cells (5 × 10^6^, resuspended in 100 μL of medium containing 50% serum-free DMEM/α-MEM and 50% Matrigel), according to a previous protocol [28]. Tumor growth in the tibiae was monitored each week by bioluminescence imaging using a Xenogen IVIS imaging system 200 (PerkinElmer, MA, USA). At 12 weeks, the mice were euthanized by CO_2_ inhalation. The lungs were removed and fixed in 10% formalin for further analysis. All animal procedures were approved and performed in accordance with the guidelines of the Institutional Animal Care and Use Committee of China Medical University (CMUIACUC-2019-079).

### IHC staining

The human chondrosarcoma tissue array OS802c was purchased from US Biomax, Inc. (Rockville, MD, USA) to collect tissue sample under the highest ethical standards, with the donors giving fully informed written consent. Study approval was granted by China Medical University Hospital’s Institutional Review Board (CMUH 107-REC3-165). Mouse lung tissues and human specimens were rehydrated and incubated with primary anti-NGF or LOX antibodies, and then treated with biotin-labeled secondary antibody. Finally, the slides were examined using the ABC Kit (Vector Laboratories, CA, USA) and photographed using the microscope. Three pictures of each slide were evaluated based on staining intensity (starting from 0: negative; 1: low; 2: weak; 3: moderate; 4: strong; and 5: very strong) conducted by three independent pathologists. The IHC score was determined as the sum of the intensity score.

### Statistical analysis

All values are presented as the mean ± standard deviation (SD). Significance testing on the difference between the groups was assessed by the Student’s *t*-test and considered significant if the *p* value was <0.05.

## Data Availability

The datasets used and analyzed during the current study are available from corresponding author on reasonable request.
